# Sex differences in colorectal cancer: with a focus on sex hormone–gut microbiome axis

**DOI:** 10.1186/s12964-024-01549-2

**Published:** 2024-03-07

**Authors:** Zihong Wu, Yuqing Huang, Renyi Zhang, Chuan Zheng, Fengming You, Min Wang, Chong Xiao, Xueke Li

**Affiliations:** 1 Hospital of Chengdu University of Traditional Chinese Medicine, Chengdu, China; 2 TCM Regulating Metabolic Diseases Key Laboratory of Sichuan Province, Hospital of Chengdu University of Traditional Chinese Medicine, Chengdu, China; 3 Institute of Oncology, Chengdu University of Traditional Chinese Medicine, Chengdu, China

**Keywords:** Colorectal cancer, Sex differences, Sex hormones, Gut microbiome, Sexual dimorphism

## Abstract

Sexual dimorphism has been observed in the incidence and prognosis of colorectal cancer (CRC), with men generally exhibiting a slightly higher incidence than women. Research suggests that this difference may be attributed to variations in sex steroid hormone levels and the gut microbiome. The gut microbiome in CRC shows variations in composition and function between the sexes, leading to the concept of ‘microgenderome’ and ‘sex hormone–gut microbiome axis.’ Conventional research indicates that estrogens, by promoting a more favorable gut microbiota, may reduce the risk of CRC. Conversely, androgens may have a direct pro-tumorigenic effect by increasing the proportion of opportunistic pathogens. The gut microbiota may also influence sex hormone levels by expressing specific enzymes or directly affecting gonadal function. However, this area remains controversial. This review aims to explore the differences in sex hormone in CRC incidence, the phenomenon of sexual dimorphism within the gut microbiome, and the intricate interplay of the sex hormone–gut microbiome axis in CRC. The objective is to gain a better understanding of these interactions and their potential clinical implications, as well as to introduce innovative approaches to CRC treatment.

**Graphical Abstract:**

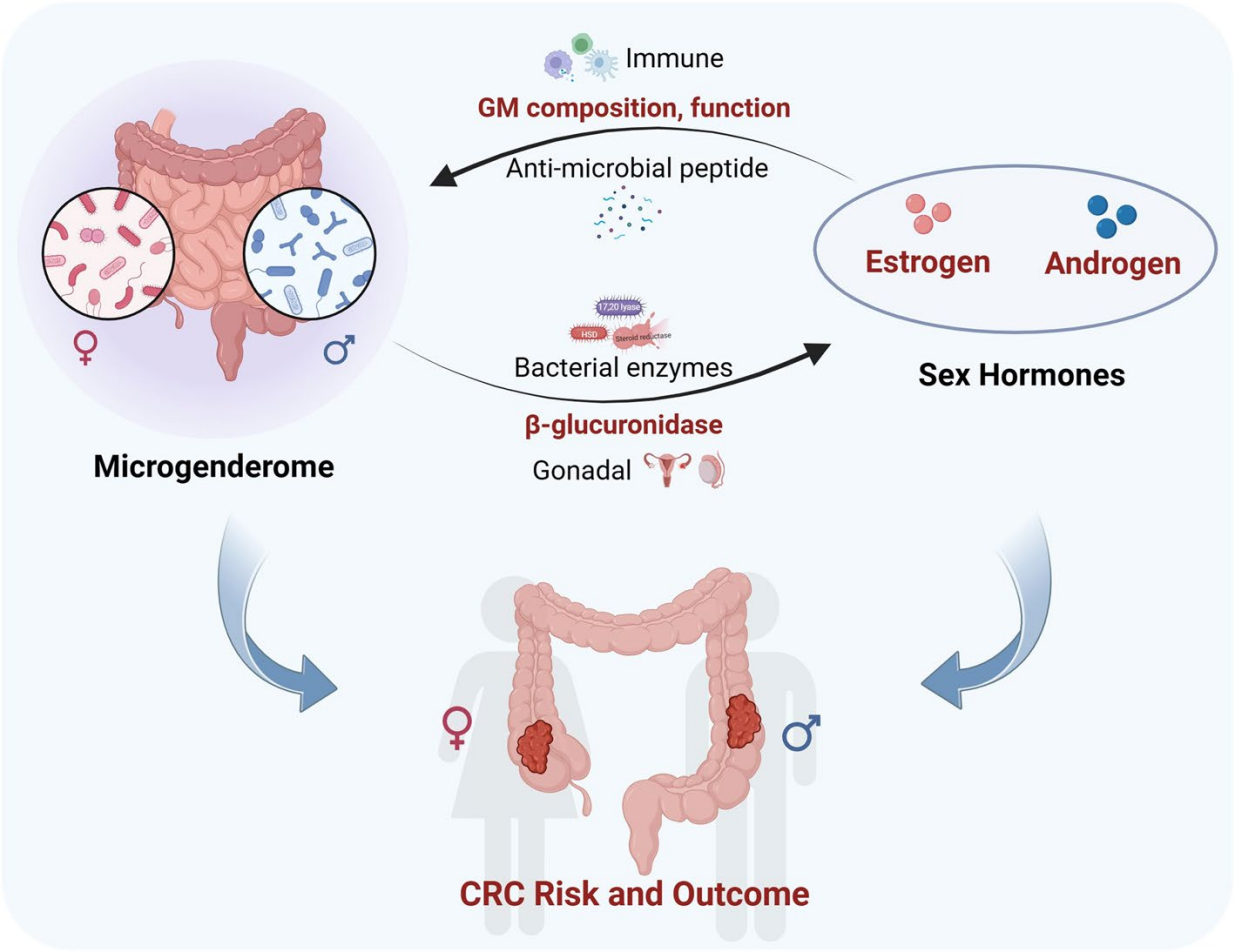

## Introduction

Colorectal cancer (CRC) represents a major public health challenge worldwide. It ranks as the third most frequently diagnosed cancer and stands as the second leading cause of cancer-related deaths in the United States. Projections for the year 2023 estimate approximately 153,020 new cases and 52,550 deaths from CRC [1]. The etiology of CRC is multifactorial, involving genetic, environmental, and lifestyle factors [2]. However, sex hormones and sex contribute to differences in CRC incidence and outcomes, underscoring the potential role of sex-specific risk factors. Recent cancer statistics have revealed that between 2015 and 2019, the average annual CRC incidence rate was 33% higher and the overall mortality rate was 43% higher in men than in women. Additionally, women exhibit a slightly higher five-year relative survival rate than men [1, 3]. These sex-specific trends can be attributed to various factors. Historically, women, particularly pregnant women, were often excluded from pivotal clinical research to protect the developing fetus from potential harm. This exclusion led to an underestimation of the influence of sex on tumor biology and clinical outcomes [4]. Moreover, dietary habits have played a role in this divergence. Men tend to consume larger quantities of meat and alcohol than women, further elevating the risk of CRC in men [5].

Recently, there has been a growing interest in the potential role of sex hormones and the gut microbiome in intestinal tumors. Previous studies have revealed an association between sex steroids and colon physiology and pathophysiology. Intestinal epithelial cells possess the ability to metabolize sex steroids, particularly estrogens, which could influence the development of CRC [6, 7]. Estrogens appear to confer protection against CRC progression, whereas androgens have been linked to an increased risk of CRC. However, controversy in this field persists, and the precise mechanisms remain unclear [4, 8]. The emerging concept of the ‘microgenderome’ underscores the interactions between sex hormones and the gut microbiota [9]. Recent investigations have indicated discernible compositional and functional differences in the gut microbiome between men and women. Generally, women exhibit higher diversity and richness of gut microbiota than men [10], and this divergence may underlie the observed sex-based differences in CRC risk and outcomes. The gut microbiome, a complex ecosystem composed of trillions of microorganisms, exerts crucial influences on host physiology, metabolism, and immune function [11]. Gut microbiota has been postulated to influence CRC development through several mechanisms, including the fermentation of undigested dietary fibers into short-chain fatty acids (SCFAs), modulation of the host immune system, and production of carcinogenic compounds [12, 13]. New evidence suggests the existence of a sex hormone–gut microbiome axis, where sex hormones regulate the composition and function of the gut microbiome and its metabolites, while the gut microbiome significantly influences sex hormone levels [10, 14].

Despite the growing evidence linking sex hormones and the gut microbiota to the development of CRC, there is a lack of research on their interactions and potential contributions to sex-based differences in CRC risk. Therefore, this review aims to summarize the current evidence on sex hormone differences in CRC incidence and the phenomenon of sexual dimorphism within the gut microbiome. Additionally, it explores the intricate interplay of the sex hormone–gut microbiome axis in CRC. The objective of this review is to better understand the interactions among sex hormones, the gut microbiome, and CRC, as well as to explore potential clinical implications and introduce innovative approaches to CRC treatment.

## Sex hormone differences in CRC

### Female sex hormones and CRC

#### Estradiol and estrone

Estrogens, primarily estradiol (E2) and estrone (E1), have been proposed as protective factors against the development of CRC. However, the relationship between estrogen and CRC remains controversial [15] (Table 1).

**Table 1:** Sex hormone-based differences in CRC

Sex hormones	Model organism	Findings	References
Estradiol/17β-estradiol (E2) Estrone	Human CRC tissue or blood sample (from postmenopausal women)	Endogenous estradiol and estrone levels are inversely associated with CRC risk and complication.	[16–23]
		Pre-diagnostic estrogen and other sex steroid levels are positively associated with mortality risk in female CRC survivors.	[34]
		Estradiol levels are not associated with CRC risk in postmenopausal women.	[36–38]
		Pathobiological role of estrogen in postmenopausal CRC varies depending on patient age and tumor characteristics.	[42]
	AOM/DSS, OVX_AOM/DSS, and OVX_Min/+ mice	Estradiol prevents colorectal carcinogenesis and metastasis by inhibiting inflammatory pathways, regulating Nrf2-related signaling, and ameliorating impaired associations with E-cadherin and β-catenin.	[26–28]
	MC38 and OVX_MC38 tumor model mice	E2 inhibits MC38 tumor growth by regulating tumor-associated cell populations and reducing PD-L1 expression. Obesity, macrophage-associated inflammation, and TAMs are potential mechanisms for inducing CRC in females lacking estrogen.	[30, 31]
		Estrogen is implicated in hepatic immunosuppression within the tumor microenvironment and promotes metastatic expansion.	[41]
	AOM-male mice DLD1, HT-29, SW480, SW620 cells and CSCs	E2 inhibits the migration and proliferation of DLD1 cells independently of miR-34a-mediated actions.	[24]
		Combined use of E2 and progesterone treatment promotes cell cycle arrest and apoptosis by stimulating the expression of ERβ and PGR and inhibiting ERα-regulated oncogenic pathways.	[32, 33]
		Combined use of E2 and 5-fluorouracil treatment exhibits superior anticancer effects than monotherapy on female and male primary CRC cells. E2 monotherapy exhibits the most substantial effects on male metastatic cells.	[33]
		E2 induces the expression of estrogen receptors on CSCs, promoting their migration and metastasis.	[40]
ERβ	Human CRC, sporadic polyps, and FAP tissue	ERβ expression is reduced in colorectal precancerous stages and plays a key role in inhibiting the development of CRC.	[44, 45, 47–49]
	AOM/DSS ERβ _KO mice	ERβ knockdown significantly induces TNFα expression and affects NF-κB inflammatory signals.	
	SW480 and HT-29 cells		
Progesterone PGR	Human CRC tissue or blood sample (from postmenopausal women)	Progesterone is generally not associated with CRC risk in postmenopausal women.	[16, 37, 38, 50]
		Progesterone and PGR expression levels positively correlate with the prognosis of CRC.	[48, 51, 52]
	Xenograft tumor model	Progesterone activates the GADD45α/JNK pathway, arrests the cell cycle, and induces apoptosis, thereby inhibiting CRC progression.	
	CRC cell lines		
Testosterone SHBG Androstenedione DHEA	CRC/adenoma patients, tissue or blood samples	Higher levels of circulating testosterone and SHBG are associated with lower CRC risk, whereas free testosterone levels are positively associated with CRC risk.	[19, 37, 54–57]
		Patients with prostate cancer who undergo androgen deprivation therapy have an increased risk of CRC	[58]
		Free testosterone levels are negatively associated with CRC incidence and mortality in both men and women.	[34, 57, 60, 61]
		Circulating concentrations of testosterone, SHBG, androstenedione, and DHEA are not associated with the risk of early precursor lesions in the colon or colon cancer	[38, 62, 63]
	AOM/DSS and ORX_AOM/DSS mice	Testosterone enhances AOM/DSS-induced CRC development.	[8, 32]
	Pirc/+ rat, Min/+ mice, and AOM mice	Sex differences in colon adenoma development may result from an indirect tumor-promoting effect of testosterone rather than a protective effect of estrogen.	[59]

*AOM/DSS *Azoxymethane/dextran sodium sulfate, *CRC *Colorectal cancer, *CSCs *Cancer stem cells, *DHEA *Dehydroepiandrosterone, *E2 *Estradiol/17β-estradiol, *ERβ *Estrogen receptor-beta, *FAP *Familial adenomatous polyposis, *OVX *Ovariectomy, *ORX *Orchiectomy, *PGR *Progesterone receptor, *SHBG *Sex hormone-binding globulin, *TIME *Tumor immune microenvironment

##### Clinical research

Clinical studies have validated that postmenopausal CRC patients have significantly lower levels of free estradiol, total estradiol, and estrone levels, as well as more advanced tumor stage compared to premenopausal CRC patients. This suggests an inverse association between estradiol and estrone levels and CRC risk [16–19]. An investigation into the expression and single nucleotide polymorphisms of the TLR4/NF-κB pathway in relation to colon cancer has found that the TLR4 *rs2770150* variant is associated with colon cancer in women over 50 years old. This association is strongly linked to the decline in female sex hormone levels in postmenopausal women [17]. Previous studies have indicated a connection between a family history of diabetes and an increased risk of CRC, and that men are more susceptible to CRC compared to women. Moreover, male patients with a family history of diabetes exhibit more significant decreases in estradiol and sex hormone-binding globulin (SHBG) levels. These associations are particularly prominent in men under 60 years old [20]. Studies have also shown that serum estradiol levels decrease by 80% after bilateral oophorectomy in premenopausal women. Women who have previously undergone oophorectomy or hysterectomy have a 24–30% higher risk of CRC compared to the general population [21, 22]. Colorectal anastomotic leakage is a common and serious complication after CRC surgery, with a higher incidence in men. Besides differences in pelvic anatomy, sex hormone differences between men and women have been suggested to play a role. Recent studies have found a lower incidence of anastomotic leakage in postmenopausal women with CRC who received preoperative estrogen replacement therapy, which may explain some of the differences in leakage rates between men and women [23]. These findings suggest a potential protective role of estrogen against CRC in women.

##### Animal and cellular models

**Fig. 1:**
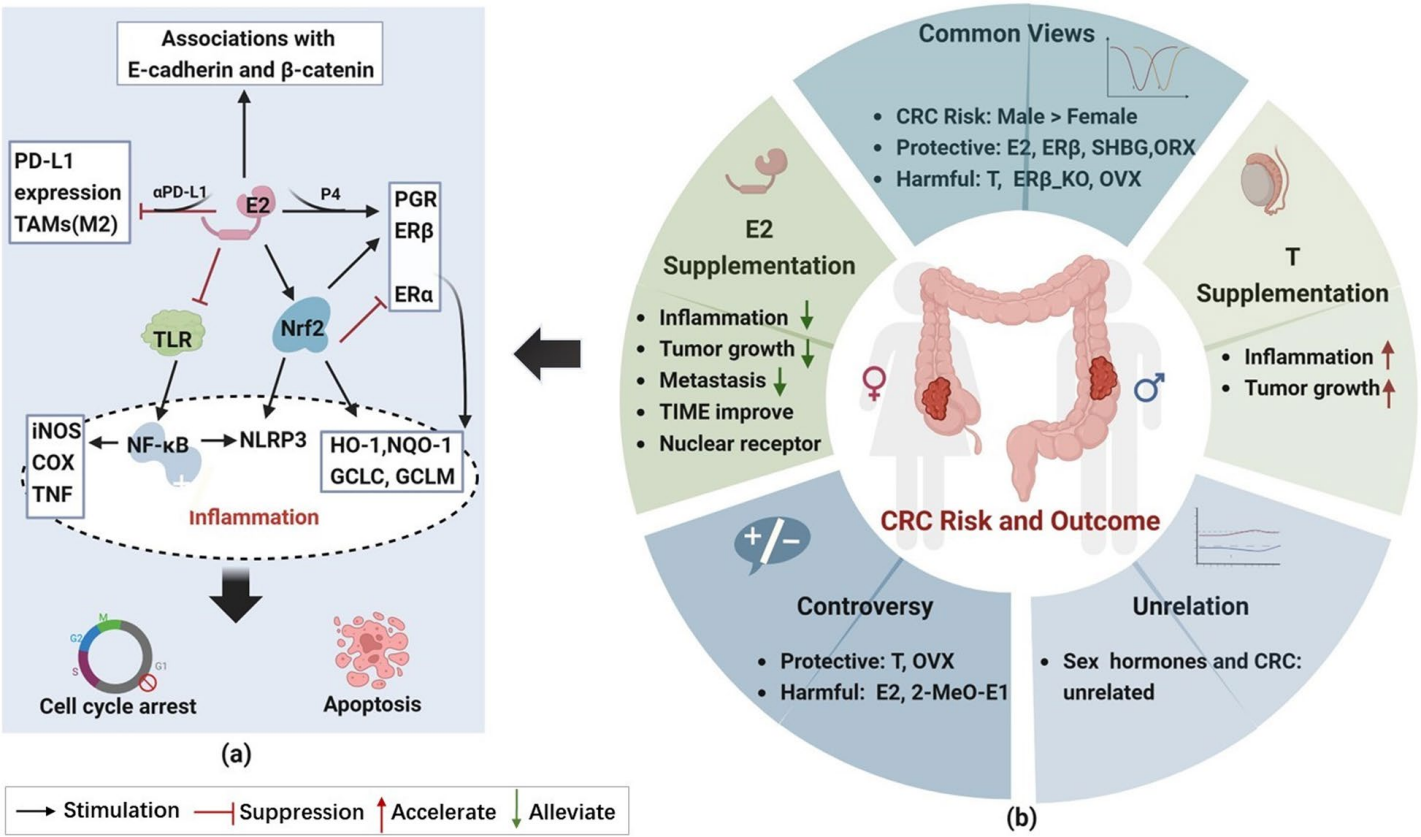
Sex hormone differences in CRC risk and outcome. **a** Molecular mechanisms underlying the protective effect of estradiol against CRC. **b** Association between sex hormones and CRC. CRC, colorectal cancer. ER, estrogen receptor. E2, Estradiol/17β-estradiol. ORX, orchiectomy. OVX, ovariectomy. PGR, progesterone receptor. P4, progesterone. SHGB, sex hormone-binding globulin. T, testosterone. TAMs, tumor-associated macrophages. TIME, Tumor immune microenvironment

In vitro experiments have demonstrated that 17β-estradiol (E2) can inhibit the migration and proliferation of DLD1 cells independently of miR-34a-mediated actions [24]. E2 has also been found to reduce inflammation in the female human colonic epithelial cell line CCD841CoN. This is achieved by decreasing the expression of inflammatory factors, such as NF-κB and COX-2, while increasing the expression of antioxidant enzymes [25]. Similarly, In the azoxymethane/dextran sodium sulfate (AOM/DSS) model, estradiol supplementation was observed to reduce the severity of colitis. Additionally, at weeks 10 and 16, male (estradiol-supplemented) and female mice had significantly lower numbers of colonic polyps and tumors compared to male mice without estradiol supplementation [26]. Furthermore, ovariectomy increases the incidence of CRC in female mouse model, but estrogen supplementation reduces the number of tumors and migration rates [27, 28]. At the molecular level, estradiol significantly increases the expression of Nrf2, which contributes to the anti-inflammatory effects of estrogen by directly regulating estrogen receptor-beta (ERβ) expression. Additionally, it upregulates Nrf2-associated antioxidant enzymes, ERβ, and NLRP3 inflammasome and downregulates the expression of ERα and NF-κB in the mouse colon. These actions ameliorate impaired associations with E-cadherin and β-catenin, ultimately inhibiting colorectal carcinogenesis and metastasis [26–29] (Fig. 1a). In the MC38 colon tumor model mice, males displayed faster colon tumor growth and a higher proportion of PD-L1 expression, M2 phenotype tumor-associated macrophages (TAMs), and cancer-associated fibroblasts than females. Furthermore, compared with anti-PD-L1 antibody alone, the combination of E2 with anti-PD-L1 antibody significantly inhibited MC38 tumor growth and decreased PD-L1 expression and M2/M1 TAM ratio. This observation suggests that E2 may synergistically enhance the anti-tumor ability of anti-PD-L1 antibodies by downregulating PD-L1 expression and regulating the number of tumor-associated cells [30]. In MC38 model mice subjected to a high-fat diet and oophorectomy, the subcutaneous adipose tissue of oophorectomized (OVX) mice exhibited more severe macrophage-associated inflammation and higher expression of M2-like genes in TAMs than that of non-OVX female mice. This observation implies that obesity, macrophage-associated inflammation, and TAMs are potential mechanisms for inducing CRC in estrogen-deficient obese women [31]. Mahbub et al. [32, 33] demonstrated that CRC is associated with abnormally altered levels of E2, progesterone (P4), and their corresponding nuclear receptors in the colon. Both monotherapy and combination therapy with E2 and P4 decreased the number of malignant lesions and ERα expression. These treatments also increased the expression of ERβ and progesterone receptor (PGR), promoting cell cycle arrest in the Sub-G1 phase and apoptosis in AOM-male mice and human male colon cancer cell lines. Additionally, the combined use of E2 and 5-fluorouracil treatment exhibited superior anticancer effects than monotherapy on female and male primary CRC cells, with E2 monotherapy demonstrating the most considerable effects on male metastatic cells (SW620). These findings confirm the potential anti-tumor effects of estrogen in males [33].

##### **Controversial findings**

Numerous studies have demonstrated the potential protective effect of estrogen against CRC; however, this relationship remains controversial. Yang et al. reported that pre-diagnosis levels of total estradiol, free estradiol, and estrone are positively associated with mortality risk in female CRC survivors [34]. Additionally, estrogen metabolite 2-MeO-E1 and COMT gene variants have been linked to CRC susceptibility in men [35]. In contrast, some studies failed to establish a significant association between estradiol levels and CRC risk [36–38]. Initial WHI trials suggested that short-term administration of estrogen plus progestin in postmenopausal women reduced CRC risk but led to more advanced-stage CRC diagnoses [39]. Consistently, an in vitro experiment demonstrated that exposure of colon cancer stem cells (CSCs) to 100 nM E2 for 48 h did not significantly affect cell viability and proliferation. However, prolonged exposure to E2 induced estrogen receptor expression on CSCs and inhibited the interaction between CSCs and HUVEC, thereby promoting CSC migration and increasing metastasis rates [40]. These results suggest that extended estrogen use may promote malignant CRC progression, ultimately increasing mortality risk. Studies in ovariectomized mice have revealed a significant reduction in CRC liver metastases, an effect augmented by estradiol supplementation. Mechanistically, estrogen deficiency results in decreased accumulation of hepatic myeloid-derived suppressor cells (MDSCs), reduced levels of immunomodulatory active proteins, and increased CD8^+^ T cell populations, all of which inhibit CRC liver metastasis [41]. To address the controversy between estrogen and CRC, a study validated the role of estrogen in CRC pathobiology based on tumor characteristics. Their findings indicated that the pathobiological role of estrogen in postmenopausal CRC varies depending on patient age, tumor location (right or left hemicolon), pathological stage, histology, E2 concentration, ERβ expression, and mismatch repair (MMR) status. Specifically, estrogen inhibits tumors characterized by L-Ca < 70 (left-sided tumors and individuals aged < 70 years), with non-medullary/mucinous (Med/Muc) histology or proficient MMR. Conversely, it promotes tumors with R-Ca ≥ 70 (right-sided tumors and individuals aged ≥ 70 years), Med/Muc histology, or deficient MMR [42]. These findings underscore the importance of considering patient age and tumor characteristics when assessing the role of estrogen in CRC.

#### ERβ

The involvement of sex hormones in the pathogenesis of CRC has been linked to ERβ, which is a nuclear receptor expressed predominantly in the colonic mucosa and ubiquitous in the intestinal tract. It primarily exerts anti-proliferative and apoptotic effects, contributing to the maintenance of normal epithelial function and tissue integrity [43]. Estrogen exerts its effects on colon cells mainly through ERβ. The expression of ERβ is reduced in human CRC tissues, adenomatous tissue, and familial adenomatous polyposis (FAP) tissues compared to normal tissues. This reduction in ERβ appears to manifest early in the development of colorectal precancerous lesions [44–48]. Survival rate seems to be more favorable in patients with ERβ-positive CRC than in those with ERβ-negative CRC, with approximately 49% of the significant CpG sites being hypermethylated in ERβ-positive tumor [44]. Furthermore, among the ERβ isoforms, the expression levels of ERβ1 and ERβ5 subtypes correlate positively with improved disease-free survival [45]. In an intestine-specific Erβ-knockout (ERβ-KO) AOM/DSS mouse model, both male and female mice exhibited increased intestinal ulcers and tumors, with male mice particularly affected. Subsequent in vitro organoid cultures and experiments with CRC cell lines validated that ERβ knockdown significantly induces TNFα expression and influences inflammatory signals such as NF-κB [47]. Naringenin, a natural estrogen disruptor, binds to ERβ. Co-treatment of HT-29 cells with naringenin and the carcinogen bisphenol A revealed that naringenin, along with its fermented extract, reduces cell viability and induces apoptosis. Mechanistically, naringenin upregulates Erβ, subsequently regulating the expression of key genes in the P53 signaling pathway. This includes the upregulation of pro-apoptotic genes, such as *FASLG* and *CASP2*, and the downregulation of anti-apoptotic genes, such as *BCL2* and *MIR141*, ultimately activating both extrinsic and intrinsic apoptosis pathways [49]. Collectively, these results suggest that intestinal ERβ plays a crucial role in repairing inflammatory damage in epithelial cells and inhibiting CRC development.

#### Progesterone and PGR

Progesterone is another female sex hormone that is critically involved in colorectal carcinogenesis, with its effects mediated through binding to the PGR. The existing literature on the association between progesterone and CRC is limited and contradictory. Some studies have reported no significant association between progesterone and CRC, and this consistency holds when analyzing colon and rectal cancer separately [16, 37, 38, 50]. Although a trend indicating an increased risk of CRC with 17-hydroxypregnenolone has been noted, it is not statistically significant [50]. However, other studies have suggested that low levels of progesterone and PGR are linked to a poorer prognosis in CRC [48, 51, 52]. Progesterone upregulates growth arrest and DNA damage‑inducible protein alpha (GADD45α), subsequently activating the JNK pathway, arresting the cell cycle, and inducing apoptosis, thereby inhibiting CRC progression [51]. Higher levels of ERβ and PGR in non-malignant tumor tissues from women aged ≤ 50 years than in men and women aged ≥ 60 years may contribute to the lower incidence of CRC in premenopausal women, as frequently reported [48]. Additionally, research has demonstrated that activation of the PGR is necessary for folic acid to inhibit the proliferation and migration of CRC cell lines [52, 53]. Collectively, these studies yield inconsistent results, warranting further investigation.

Briefly, most studies substantiate that estrogen exerts a protective effect against CRC in both men and women. A significant decline in estrogen levels in postmenopausal women is strongly linked to an elevated risk of CRC. Mechanistically, estrogen promotes cell cycle arrest and apoptosis primarily by inhibiting the NF-κB inflammatory pathway and tumor-associated cell populations, particularly M2-type macrophages and PD-L1-expressing cells. It regulates Nrf2-associated signaling, upregulates ERβ and PGR, and suppresses ERα-regulated oncogenic pathways, thus preventing CRC development and progression. However, some studies have suggested a positive association between estrogen and CRC risk, suggesting that prolonged estrogen use may promote malignant progression. The pathobiological role of estrogen in postmenopausal CRC varies depending on patient age and tumor background. Conversely, the relationship between progesterone and CRC risk lacks significance, and the evidence remains insufficient. Therefore, the association of estrogen and progesterone with CRC is controversial. The duration of sex hormone use, patient age, and tumor characteristics must all be comprehensively considered when analyzing the role of female sex hormones in CRC (Fig. 1b).

### Androgen and CRC

Androgen has been increasingly linked to CRC (Table 1). Numerous clinical studies have shown that higher levels of circulating testosterone and SHBG are associated with a decreased risk of CRC, while elevated free testosterone levels are positively correlated with CRC risk [19, 37, 54–57]. In addition, patients with prostate cancer who undergo androgen deprivation therapy have an increased risk of CRC, particularly distal colon adenocarcinoma [58]. In an AOM/DSS-induced colitis-associated cancer model, serum testosterone levels were significantly increased in orchiectomized (ORX) male and female mice after AOM/DSS treatment compared with those in controls. Orchiectomy (ORX) significantly reduced the incidence of colitis and distal colonic tumors in male mice. When supplemented with testosterone, the expression of inflammatory mediators COX-2 and iNOS increased, exacerbating colonic inflammation and greatly contributing to the development of submucosal invasive carcinoma [8]. Previous studies have confirmed that certain animal models of colon cancer, including Apc^*Pirc/+*^ rats, Apc^*Min/+*^ mice, and AOM-mice, exhibit increased male susceptibility to colon adenoma development [32, 59]. The reduction in endogenous hormone levels after ovariectomy did not alter the prevalence of adenomas in females, whereas androgen depletion during orchiectomy significantly inhibited adenoma development in Apc^*Pirc/+*^ rats and AOM-mice. Furthermore, androgen receptors were not detected in the colon and adenomas, suggesting that the sex difference in colon adenoma development may be an indirect tumor-promoting effect of testosterone rather than a protective effect of estrogen [59].

Conversely, some studies suggest that androgens may have a protective effect against CRC [34, 57, 60, 61]. Both men and women showed a negative correlation between free testosterone levels and CRC morbidity and mortality [34, 57]. In men, the risk of CRC was increased due to obesity-induced reductions in SHBG and testosterone levels [60]. Additionally, testosterone therapy was found to be negatively associated with distant-stage CRC [61]. Mori et al. [37] suggested that the association between SHBG levels and CRC risk may vary depending on total isoflavone intake. However, several studies have reported that circulating concentrations of testosterone, SHBG, androstenedione, and dehydroepiandrosterone (DHEA) are not associated with early precursor lesions of the colon or the risk of colon cancer [38, 62, 63].

In summary, sex differences in CRC risk and outcomes exist, and sex hormones, primarily estrogen and testosterone, are thought to play a significant role in this sex dimorphism. Although the relationship between sex hormones and CRC risk remains controversial, existing studies can provide partial insights into the sex dimorphism observed in CRC (Fig. 1). Considering the strong association between the gut microbiome and CRC development, coupled with the bidirectional interaction between sex hormones and the gut microbiome, we will further explore sexual dimorphism in the gut microbiome in CRC in the next section.

## Sexual dimorphisms in the gut microbiome in CRC

### Influence of the gut microbiome on CRC

The gut microbiome is made up of approximately 10^13^–10^14^ microorganisms that play a role in regulating host metabolism, inflammatory responses, immune system, and intestinal barrier function. Research has shown that the composition of the gut microbiome is strongly influenced by sex and age [9, 64]. In older adults, there is a decrease in *Bifidobacteria* and *Lactobacilli*, and a significant increase in *Bacteroidetes* and *E. coli* in the intestinal bacteria [65]. Among the primary microbial phyla in the gut microbiota, *Firmicutes* (F) and *Bacteroidetes* (B) are particularly important, and the F/B ratio is used as an indicator of the overall balance of the gut microbiota [66]. An elevated F/B ratio is generally associated with obesity and dietary habits, while a reduced F/B ratio is typically observed in cases of inflammatory bowel disease. However, the evidence for a correlation between this ratio and these diseases is not yet sufficient [66, 67].

In recent years, it has been increasingly recognized that the gut microbiome is involved in CRC development [13, 68, 69]. It has been shown that ecological dysregulation of the gut microbiome is a risk factor for CRC [70]. Previous studies have indicated that the F/B ratio increases from birth to adulthood and then gradually decreases with age [65]. However, new evidence suggests that the F/B ratio sequentially increases in younger volunteers, older volunteers, and older CRC patients [71]. These findings indicate that the same bacteria may have different functions in different populations. CRC is more prevalent in middle-aged and elderly populations, and specific bacteria colonizing the gut of young people may have a beneficial role in preventing inflammation and tumorigenesis. On the other hand, bacteria enriched in the guts of older individuals may promote colorectal tumorigenesis. At the phylum level, the dominant genera in CRC include *Bacteroidetes*, *Faecalibacterium*, *E. coli*, *Fusobacterium nucleatum*, *Streptococcus gallolyticus*, and *Peptostreptococcu*s [72–74]. Several studies have shown that bacteria such as *enterotoxigenic Bacteroides fragilis*, *E. coli*, and *Fusobacterium nucleatum* are involved in the development of chronic colitis-associated cancers [74–76]. Moreover, higher abundance of *Bacteroidetes* has been associated with chemotherapy-related adverse effects and poorer prognosis [77, 78]. Additionally, among patients treated with immune checkpoint inhibitors, those with high abundance of *Bacteroidetes* have shown shortened progression-free survival compared to those with low abundance [78, 79]. Mechanistically, patients with relatively high abundance of *Bacteroidetes* exhibit limited intratumoral lymphocyte and myeloid cell infiltration, as well as diminished antigen-presenting capacity, resulting in impaired systemic and antitumor immunity [78]. These findings highlight the significant contribution of intestinal bacteria, particularly *Bacteroidetes*, in the progression of CRC and the potential for therapeutic intervention by modulating the gut microbiome in patients undergoing immune checkpoint inhibitor treatment.

### Sex differences in the gut microbiome in clinical samples

Recent studies have highlighted differences in the gut microbiome composition between males and females [80–85]. These differences may contribute to the observed variations in CRC risk and outcomes between the sexes. To investigate the correlation between intestinal flora and sex, Lin et al. [80] generated a heatmap based on the sex of CRC patients. They discovered that females had higher levels of five bacterial species, including *Prevotella* sp. Marseille-P2931, *Clostridium colinum*, and *Bifidobacterium pseudocatenulatum*. On the other hand, male patients showed significant enrichment of 11 bacterial species, including *Fusobacterium mortiferum*, *Bifidobacterium adolescentis*, and *Succinatimonas hippei*. Another clinical study confirmed that *Bacteroides* are important bacteria associated with CRC [81]. In male CRC patients, the most detected bacteria were *Bacteroides*, *Eubacterium*, and *Faecalibacterium*, while in female patients, they were *Bacteroides*, *Subdoligranulum*, and *Eubacterium*. Additionally, *Blautia*, *Barnesiella*, and *Anaerostipes* were identified as the three bacteria that differed the most between male and female patients [81]. Liao et al. [82] also observed significant variations in microbial diversity, community structure, and microbial symbiosis between males and females during CRC development. They found that males had more stable gut microbial communities compared to females. As CRC advanced, the gut microbial β-diversity increased in males, without significant changes in α-diversity, resulting in more stochastic gut microbial communities with complex microbial symbioses. Conversely, both α- and β-diversity decreased significantly in females, leading to more deterministic microbial communities but with the absence of key species [82]. Previous research has demonstrated that *Escherichia coli* containing polyketide synthase (*pks* ^*+*^ *E. coli*) can promote the progression of CRC. In a cross-sectional study investigating the relationship between dietary intake and the prevalence of *pks* ^*+*^ *E. coli*, it was found that men had a significantly higher prevalence compared to women. This disparity may partially explain the difference in CRC incidence between the sexes [83]. A recent study discovered that healthy men had lower levels of intestinal lactobacilli and butyrate-producing bacteria compared to healthy women. However, these sex-related differences were not observed in patients with colonic adenomas and CRC. This suggests that the absence of certain probiotics could contribute to the higher incidence of CRC in men [85].

The gut microbiome in CRC not only exhibits sexual dimorphism in its composition and function but also influences sex-specific aspects of preventive and therapeutic approaches based on intestinal microbiome. For example, serum vitamin D levels are negatively associated with CRC risk and prognosis. Female patients with CRC are more susceptible to *Fusarium nucleatum* infections following vitamin D supplementation than their male counterparts [84]. Secondary bile acids, such as deoxycholic acid (DCA), are tumor-promoting bile acids produced by anaerobic colonic bacteria via 7α-dehydroxylation. Ursodeoxycholic acid (UDCA) mitigates the effects of DCA and inhibits colon cancer activity in mouse models [86]. In patients treated with UDCA, an increased abundance of *Faecalibacterium prausnitzii* and a deficiency of *Ruminococcus gnavus* were significantly associated with a higher risk of colon adenomas in men. However, such associations were not observed in women. These results imply that sex alters the activity of UDCA in the colon [87].

### Sexual dimorphism in the gut microbiome of animal models

#### Sex differences

The sex-biased gut microbiome in CRC is also prevalent in animal models. Previous studies have indicated the presence of sex dimorphism in the epigenetic genes of the colons of prepubescent mice. However, these studies did not identify significant sex-based differences in the composition of the gut microbiome; rather, they suggested that sex primarily influences the dominance of specific taxa [88]. Recent investigations have revealed that Apc^Min/+^ and AOM/DSS male mice exhibit larger and more numerous colon tumors and greater severity of gut inflammation than female mice. Additionally, in male and pseudo-germ mice that received fecal samples from male mice or men, the intestinal pathogenic bacterium *Akkermansia muciniphila* was significantly enriched. Conversely, the abundance of the probiotic bacterium *Parabacteroides goldsteinii* was significantly reduced, leading to a deterioration in intestinal barrier function. Mechanistically, the predominant male-associated intestinal microbial metabolites activate the glycerophospholipid metabolic pathway, ultimately exacerbating CRC tumorigenesis. Therefore, the modulation of sex-biased gut microbiomes and associated metabolites may represent a potentially effective, sex-targeted strategy for both the prevention and treatment of CRC [89].

#### Microgenderome

The “microgenderome,” referring to interactions between sex hormones and the gut microbiome, is an emerging area of study. Research data from various animal models have established the role of the “microgenderome” in CRC incidence. Song et al. [27] discovered that E2 inhibits AOM/DSS-induced tumorigenesis in male mice. They conducted a study to further investigated the effects of E2 on the gut microbiome. They found that E2 supplementation in males, AOM/DSS + E2 treatment in males and females, led to a significant increase in microbial diversity compared to normal male mice. This increase was indicated by OTU counts and the Chao 1 index. Furthermore, they observed a significant reduction in *Bacteroides* abundance and F/B ratio in AOM/DSS + E2 male mice compared to AOM/DSS mice [90]. *Bacteroides* are major promoters and facilitators of human CRC and have the potential to exacerbate tumorigenesis in the AOM/DSS model [76, 91]. Additionally, the ratio of commensal bacteria to opportunistic pathogens (C/O) was higher in E2-supplemented males and females than in normal males and females_OVX [90]. Building upon their discovery that E2 can inhibit MC38 tumor growth by reducing PD-L1 expression, Song et al. also found that E2 pretreatment prior to anti-PD-L1 treatment induced changes in the intestinal microbiota of MC38 mice. These changes included a decrease in the abundance of the *Muribaculaceae* family and opportunistic pathogens (*Enterobacteriaceae* group) and an increase in the abundance of the *Ruminococcaceae* family and commensal bacteria (*Lactobacillus murinus* group and *P. goldsteinii*). These alterations may contribute to enhanced anti-tumor immunotherapy [92]. These findings indicate that estradiol has the ability to induce changes in the gut microbiome of colon tumor model mice. These changes include a decrease in the abundance of *Bacteroides* and the F/B ratio, an increase in the C/O ratio, and alterations in microbiota diversity. These alterations are closely linked to an improvement in anti-tumor immunity and a lower risk of CRC [90, 92].

Intestinal ERβ serves as a protective factor against colitis-associated cancers. Studies have shown that colitis-induced CRC reduces gut microbiota diversity and enriches gram-negative bacteria, particularly in the absence of ERβ. In AOM/DSS ERβ-KO mice, males exhibited an enriched microbiota with functions related to cell motility, membrane transport, and carbohydrate metabolism, whereas females exhibited a reduced microbiota with functions primarily associated with metabolism and the endocrine system. This observation suggests that ERβ facilitates a more favorable gut microbiome, which, in turn, may contribute to the prevention of CRC development [93]. Prior research has demonstrated that Nrf2 can enhance the anti-inflammatory effects of estrogen by directly regulating ERβ expression. Recent studies have also indicated that Nrf2 genotypes can alter gut microbiome composition. In AOM/DSS Nrf2_KO mice, changes in the abundance of *Akkermansia muciniophila*, *L. murinus*, and *Bacteroides vulgatus* were observed. These changes varied between sexes and the extent of CRC induction. Specifically, *B. vulgatus* abundance increased in both male and female AOM/DSS mice, whereas *L. murinus* abundance was decreased only in Nrf2_KO male mice. Furthermore, the abundance of *(A) muciniophila* increased in male mice, regardless of Nrf2 knockout. Here, *L. murinus* abundance was negatively correlated with the number of colon tumors, whereas *(B) vulgatus* abundance was positively correlated with inflammatory status, tumor count, and adenoma grade [94]. These findings suggest that Nrf2 may reduce the incidence and progression of colitis-associated cancers by regulating ERβ expression and shaping a more favorable gut microbiota.

Recent reports suggest that the dysregulation of the intestinal microecology induced by testosterone may play a crucial role in the differences in colorectal carcinogenesis between sexes [95]. In the control group, male mice exhibited higher levels of *Firmicutes*, lower levels of *Bacteroidetes*, and higher F/B ratios compared to female and ORX mice. In the AOM/DSS and AOM/DSS + testosterone propionate (TP) groups, there were no significant differences in F/B ratios between females and males. However, in the ORX AOM/DSS group, the addition of TP did not result in a difference in *Bacteroidetes*, but it led to a decrease in the abundance of *Firmicutes*, which in turn decreased the F/B ratio. Additionally, a decline in microbial diversity and the C/O ratio was observed [95]. Testosterone supplementation increased the probability of infection with opportunistic pathogens (*Mucispirillum schaedleri* or *A. muciniphila*) in AOM/DSS females and ORX mice [95].

#### Microgenderome-based therapeutic approaches

Sexual dimorphism in gut flora plays a role in CRC treatment. Recent studies have revealed that anti-PD-L1 treatment reduces testosterone levels in male MC38 mice while significantly altering the composition of the gut microbiome in female mice [96]. When male mice were treated with narrow-spectrum antibiotics, they exhibited a more robust recovery of microbiota dysbiosis index (MDI), improved colitis, mitigation of testicular lipid metabolism disorders, and enhanced efficacy of anti-PD-L1 immunotherapy efficacy compared with those in female mice. At the genus level, the abundance of *Lachnospiraceae* was significantly lower in male mice than in females, whereas the converse was true for the *Muribaculaceae* family [96, 97]. *Lachnospiraceae* has been linked to immune checkpoint inhibitor responses [98], whereas *Muribaculaceae* plays a key role in AOM/DSS-induced CRC [99]. These findings underscore the potential significance of sex hormones as targets for enhancing the anti-tumor efficacy of anti-PD-L1. Moreover, they highlight the importance of considering sex differences in the gut microbiome when using antibiotics to manage immune checkpoint inhibitor-associated colitis.

Zearalenone (ZEA), an estrogenic mycotoxin, has shown promise in inhibiting colitis-associated colorectal tumorigenesis in male mice. It achieves this by increasing the abundance of SCFA-producing bacteria like unidentified *Ruminococcaceae*, *Blautia*, and *Parabacteroidies* while concurrently inhibiting the RAS/RAF/ERK pathway [100]. Additionally, the weakly estrogenic compound isoxanthohumol, along with its intestinal microbial metabolite 8-PN, demonstrates the ability to disrupt the cell cycle and inhibit the proliferation and invasion of colon cancer cells [101]. *Helicobacter* spp. is suspected to act as a deleterious stimulus, creating an environment of chronic intestinal inflammation that drives CRC development. Wolfe et al. uncovered complex dynamic changes in the intestinal microbiome when mice inoculated with the Th17-enhanced commensal candidate *Candidatus Savagella*, referred to as segmented filamentous bacteria (SFB), were exposed to *Helicobacter* spp. SFB^+^ male mice exhibited a significantly lower incidence of CRC than SFB^+^ female mice. The *Enterobacteriaceae* family, which ultimately developed CRC, was significantly increased in abundance exclusively in SFB^+^ mice. Moreover, the relative abundance of *Enterobacteriaceae* was higher in female mice than in male mice. These results suggest that SFB stabilizes the transgene against changes induced by *Helicobacter* spp. following inoculation and exerts a sex-dependent protective effect in male mice [102].

**Fig. 2:**
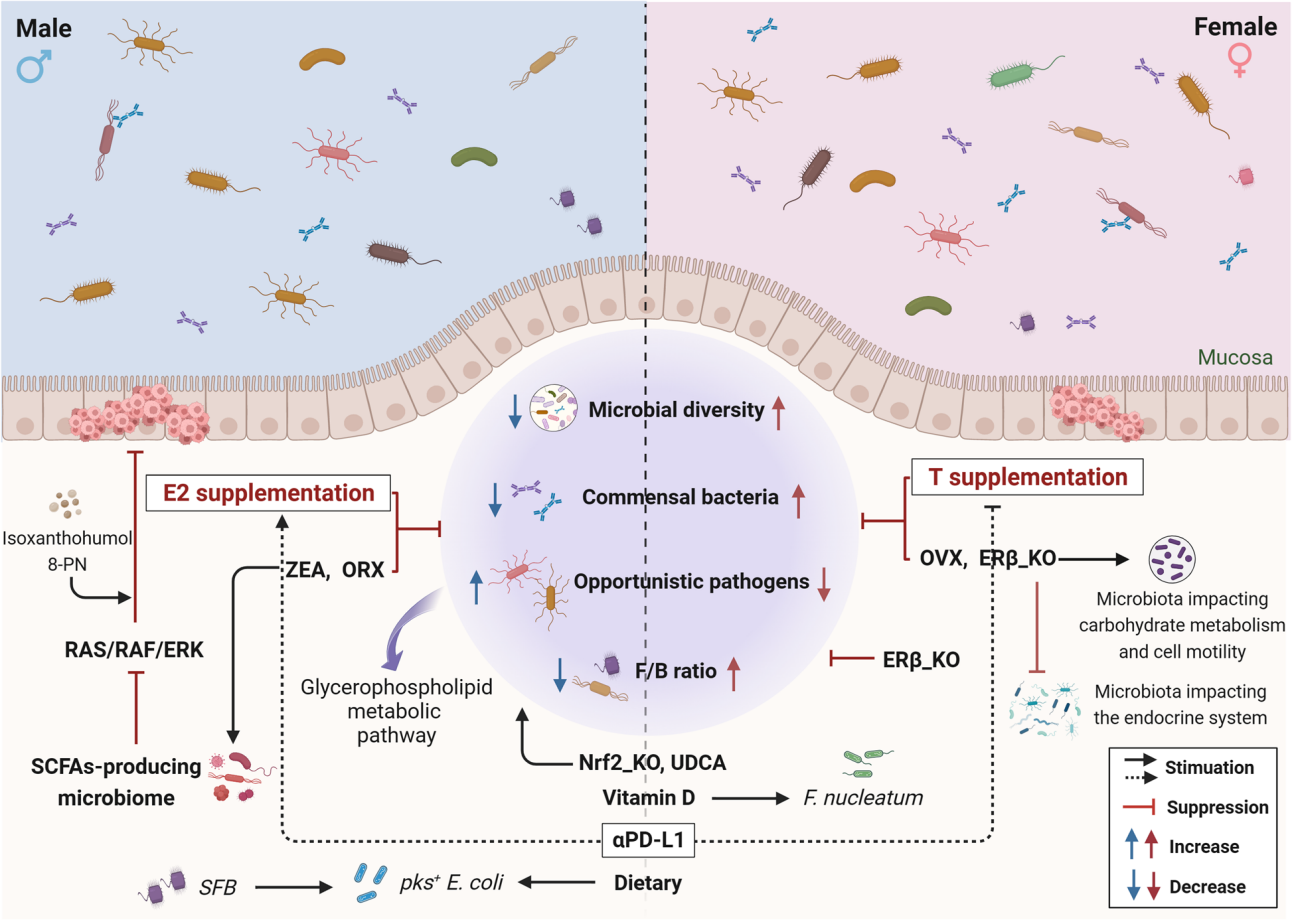
Sexual dimorphism in the gut microbiome in CRC. CRC, colorectal cancer. ERβ, estrogen receptor-beta. E2, Estradiol/17β-estradiol. ORX, orchiectomy. OVX, ovariectomy. SCFAs, short chain fatty acids. SFB, Segmented Filamentous Bacteria. T, testosterone. ZEA, Zearalenone

Collectively, the gut microbiome in CRC is sexually dimorphic in composition, function, and microbe-based therapeutics. The interplay among between sex, sex hormones, gut microbiota, and CRC is intricate and multifaceted (Fig. 2). In broad terms, estrogen and ERβ may mitigate CRC risk by fostering a more favorable gut microbiota. This includes altering the F/B ratio, increasing the C/O ratio, and enriching microbiota diversity, thereby enhancing anti-tumor immunity. Conversely, testosterone-induced dysregulation in the intestinal microecological may constitute a pivotal factor contributing to sex-based differences in colorectal carcinogenesis (Table 2). Understanding these relationships can aid in identifying individuals at risk for CRC development and optimizing treatment strategies for this disease. In the next section, we will delve further into the mechanisms governing the interaction between sex hormones and the gut microbiome.

**Table 2:** Summary of sexual dimorphism in the gut microbiome in CRC

Model organism	Sex dimorphism	Change in Gut microbiome		Findings	References
		Increase (Enrich)	Reduce		
CRC/adenoma patients	male	*F.mortiferum, B. adolescentis, S. hippei, P. gingivalis, A. intestini, Clostridium sp.* AT4, *D. propionicifacien, M. smithii, B. massiliensis, F. varium, L. bacterium*		The composition and relative abundance of the gut microbiome in CRC would be influenced by sex	[80]
	female	*Prevotella sp. Marseille*, *C. colinum, B. pseudocatenulatum, Gordonibacter sp. Marseille*, *C. Saccharibacteria*			
	male	*Bacteroides*, *Eubacterium*, *Faecalibacterium*			[81]
	female	*Bacteroides, Subdoligranulum, Eubacterium*			
CRC/adenoma patients	male	microbial β-diversity, rare species, more stochastic community structure		The gut microbial communities are more stable in males than in females during the development of CRC	[82]
	female	more deterministic community structure	microbial α- and β-diversity, key species		
Patients with pks + E. coli	male	*pks + E. coli*		The prevalence of *pks + E. coli* was significantly higher in men than women	[83]
	female	*pks + E. coli*			
CRC patients	female	*F. nucleatum*		Women are more susceptible to *F. nucleatum* following Vitamin D supplementation	[84]
Adenoma patients	male	*F. prausnitzii*	*R. gnavus*	Gender alters UDCA activity in the CRC	[87]
	female	no difference			
Min/+ and AOM/DSS mice	male	*C.aerofaciens, D. bacterium, A. muciniphila, A. inops*	*P. goldsteinii, L.taiwanensis, L. fermentum*	Gender-biased gut microbiome and metabolites favor sex dimorphism in CRC	[89]
	female	microbial diversity, beneficial bacteria	harmful bacteria		
AOM/DSS mice	E2-treated male	microbial diversity	*Bacteroides*, F/B ratio	E2 may induce changes in the gut microbiota, thereby reducing the risk of CRC	[90]
	male	C/O ratio			
	female_OVX		C/O ratio		
MC38 tumor model mice	αPD-L1 + E2 male	family *Ruminococcaceae*, *L. murinus* group and *P. goldsteinii*	family *Muribaculaceae*, Enterobacteriaceae group	E2 pre-treatment prior to anti-PD-L1 therapy induces changes in the gut microbiome of MC38 mice, thereby contributing to anti-tumor therapy	[92]
	αPD-L1 male	*P. goldsteinii*			
	αPD-L1 female				
AOM/DSS mice	male	*Parasutterell*a	microbiota diversity	ERβ facilitates a more favorable gut microbiome, which may prevent the development of CRC	[93]
	fenale	*Prevotellaceae*_UCG_001	microbiota diversity		
	ERβ_KO	*Rikenellaceae*_RC9; *Lachnospiracae*_UCG_010	microbiota α- diversity, Chao-1index		
AOM/DSS mice	male	*A. muciniophila; B. vulgatus*	microbiota α- diversity	Alterations in intestinal flora composition by Nrf2 depend on gender and CRC induction	[94]
	female	*B.* abundance	*L. murinus*		
	male Nrf2_KO	*A. muciniophila*	microbial α-diversity, L. murinus		
	female Nrf2_ KO	No difference			
AOM/DSS mice	male_ORX	microbial diversity; F/B ratio		Testosterone-induced dysbiosis of the gut microbiota may be a factor in sex differences in colorectal carcinogenesis	[95]
	Male_ORX + TP		microbial diversity, F/B ratio; C/O ratio		
	TP- male and female	opportunistic pathogens (*M. schaedleri* or *A. muciniphila*)	C/O ratio		
	female	microbial diversity, beneficial bacteria	harmful bacteria		
MC38 tumor model mice	male	Family *Muribaculaceae*	microbial α-diversity, *Lachnospiraceae* group	Sex differences in the gut microbiota should be considered when applying antibiotics for the treatment of immune checkpoint inhibitor-associated colitis	[96, 97]
	female	*Lachnospiraceae* group	microbial α-diversity, Family *Muribaculaceae*		
AOM/DSS mice	ZEA^+^ male	unidentified *Ruminococcaceae*, *Parabacteroidies*, *Blautia;* microbial community stability	Microbial community vulnerability	Zearalenone increases SCFAs-producing intestinal microbiome with good inhibitory effects on CRC	[100]
Smad3^−/−^ CAC model	SFB^+^male	*Helicobacter* spp., SFB		SFB has a sex-dependent protective effect in CRC male mice	[102]
	SFB^+^female	*Helicobacter* spp., SFB, family *Enterobacteriaceae*			

*AOM/DSS *Azoxymethane/dextran sodium sulfate, *CRC *Colorectal cancer, *C/O *Commensal bacteria/opportunistic pathogens, *E2 *Estradiol/17β-estradiol, *ERβ *Estrogen receptor-beta, *F/B *Firmicutes/Bacteroidetes, *OVX *Ovariectomy, *ORX *Orchiectomy, *pks + E*. coli Escherichia coli containing polyketide synthase, *SCFAs *Short chain fatty acids, *SFB *Segmented Filamentous Bacteria, *TP *Testosterone propionate, *UDCA *Ursodeoxycholic acid, *ZEA *Zearalenone

## Sex hormone–gut microbiome axis and its role in CRC

**Fig. 3:**
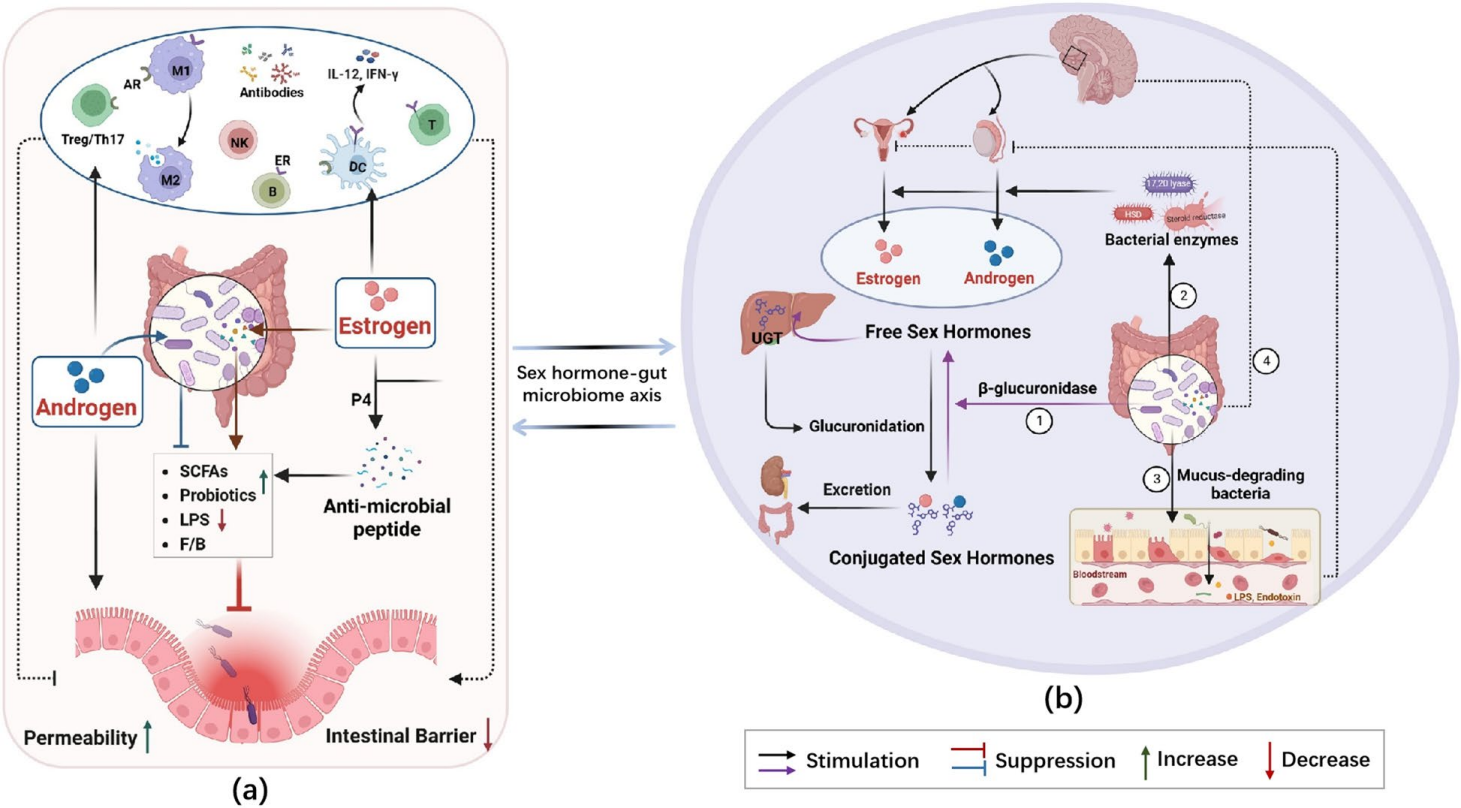
Sex hormone–gut microbiome axis and its role in CRC. **a** Influence of sex hormones on the composition and function of the gut microbiome. **b** Potential mechanisms through which the gut microbiome regulates sex hormone levels. AR, androgen receptor. CRC, colorectal cancer. ER, estrogen receptor. P4, progesterone. UGT, UDP-glucuronosyltransferase

Generalizing the differences in the gut microbiome based on sex is challenging due to variations in study findings. However, researchers had identified several gut flora that were related to sex hormones. Emerging evidence supports the existence of the sex hormone–gut microbiome axis, which describes a bidirectional interaction between sex hormones and the gut microbiome. This interaction suggests that sex hormones can influence the composition and function of the gut microbiome and its metabolites, while the gut microbiome can also have a significant impact on sex hormone levels [9, 69] (Fig. 3).

### Influence of sex hormones on the gut microbiome

The sex hormone–gut microbiome axis operates through multiple mechanisms (Fig. 3a). One important mechanism is the regulation of the gut microbiota composition by sex hormones. Estrogen, a major regulator of the gut microbiota, modulates the composition of gut microbiome by promoting the growth of bacteria that produce SCFAs (e.g., butyrate). These SCFAs play a role in inhibiting inflammatory signaling pathways, enhancing gut barrier function, and improving energy metabolism [92, 103, 104]. Progesterone supplementation increases the relative abundance of intestinal *Bifidobacterium* in women and mice during late pregnancy [105]. Conversely, testosterone increases the abundance of *Firmicutes* and decreases that of *Bacteroidetes* [95, 106, 107]. A study found that individuals with high estradiol or testosterone levels exhibited highly diverse gut microbiota. In men, testosterone levels positively correlated with the abundance of *Ruminococcus*, *Acinetobacter*, *Megamonas*, and *Dorea*. Conversely, women with higher estradiol levels exhibited a higher *Bacteroidetes* abundance and a lower *Firmicutes* abundance [108].

Sex hormones can also influence the immune response through the gut microbiota, which plays a crucial role in the development and function of the immune systemn. Commensal bacteria support the maturation of the intestinal mucosal immune system, while pathogenic bacteria can cause immune dysfunction [109]. Sex hormone receptors are widely expressed in immune cells, allowing sex hormones to directly regulate bacterial metabolism via these receptors [110]. Animal studies have revealed that E2 and estrogen-like compounds enhance the prevention of low-grade intestinal chronic inflammation by upregulating the expression and activity of intestinal alkaline phosphatase (IAP), an intestinal microbial-regulated anti-microbial peptide. This reduces the abundance of *Proteobacteria* and lipopolysaccharide biosynthesis [111]. Knockdown of ERβ in female mice disrupts the gut microbiota, enriching microbes that affect the immune system [93, 112]. Additionally, the gut microbiota influences T-cell development and antibody production in a sex-dependent manner [113]. However, earlier studies suggested that β-estradiol promotes IL-12 and IFN-γ production by dendritic cells, activating pro-inflammatory pathways. It also prolongs B-cell survival, creating an inflammatory microenvironment with altered intestinal permeability, which leads to the migration of gut microbiota into the lamina propria, promoting inflammation [9]. Androgens are generally considered immunosuppressive, but some studies have found that testosterone and DHEA promote the activation of regulatory T cells, thereby suppressing Th17-type responses [114]. Others have demonstrated that testosterone inhibits T-cell proliferation, modulates macrophage function, and promotes inflammatory responses [115]. These seemingly contradictory results align with previous research indicating that the roles of sex hormones and the gut microbiome in the development of CRC are intricate and diverse. Consequently, further studies are required to validate these findings.

### Potential mechanisms through which the gut microbiome regulates sex hormone levels

Sex hormones have the ability to impact the composition and diversity of the gut microbiome. This, in turn, plays a role in regulating the excretion and cycling of sex hormones. The gut microbiome can regulate host sex hormone levels through a variety of potential mechanisms (Fig. 3b). Firstly, specific enzymes called UDP-glucuronosyltransferases (UGTs) in the liver catalyze the glucuronidation of sex hormones, which are then excreted into the urine or eliminated through the bile duct into the intestine [116]. These conjugated estrogens and androgens are deconjugated by β-glucuronidase, which is an enzyme responsible for breaking down sex hormones into their active forms. This process results in the formation of biologically active free sex hormones that are reabsorbed through the enterohepatic circulation, ultimately influencing downstream physiological effects. Dysregulation of the intestinal microbiota can lead to reduced deconjugation, resulting in decreased levels of free estrogens [103, 117–119]. The gut microbiome can metabolize both endogenous and exogenous estrogens, with the metabolites produced having an impact on the host. When estrogen-metabolizing bacteria are deficient, circulating estrogen levels decrease, which may be a risk factor for certain conditions, including cancer. The gut microbiota is a crucial regulator of androgen metabolism. Compared with female mice, male mice exhibit significantly elevated β-glucuronidase activity, high concentrations of glucuronidase testosterone and dihydrotestosterone (DHT) in their small intestinal contents, and increased levels of free DHT in the distal intestine or feces. However, germ-free mice show low levels of free DHT in the distal intestine. These findings suggest that the gut microbiota influences the intestinal metabolism of testosterone and DHT [9, 117].

Secondly, emerging evidence strongly supports the potential involvement of the gut microbiota in the biosynthesis of various sex steroid hormones. This is primarily achieved through the activity of specific bacterial enzymes, including 17, 20 lyase, hydroxysteroid dehydrogenase (HSD), and steroid reductase [120, 121]. However, the available evidence on bacterial enzymes is currently limited, and further research is required to confirm these findings. Additionally, human fecal bacteria can oxidize, reduce, and hydrolyze estrogens and androgens [14]. Thirdly, the gut microbiome may influence sex hormone levels by directly affecting gonadal function. Certain mucus-degrading bacteria play a role in maintaining mucus function. Disruption of the gut mucosal barrier can facilitate the entry of microbiota from the intestinal lumen into the circulation, potentially inducing systemic chronic inflammation. Such inflammation can inhibit testosterone production by testicular cells [14, 122]. In contrast, some intestinal microorganisms are involved in regulating the secretion of neuromodulators in the gut–brain axis, which can influence the production of endogenous androgens and estrogens via the hypothalamic–pituitary–testis or ovary axis [123, 124].

### Role of the sex hormone–gut microbiome axis in CRC

Sex steroid hormones, particularly estrogen and testosterone, are believed to play a pivotal role in the sexual dimorphism of CRC risk and outcome. Extensive research consistently shows that estrogen has a protective effect against CRC in both men and women. Conversely, higher levels of free testosterone are associated with an increased risk of CRC. Recent studies have identified differences in the gut microbiome between males and females in the context of CRC. New evidence now supports the existence of a sex hormone–gut microbiome axis, which may explain the observed disparities in CRC risk and outcomes between the sexes. Mechanistically, estrogen enhances anti-tumor immunity by promoting a more favorable gut microbiota. This involves increasing the C/O ratio and enriching microbiota diversity by inhibiting inflammatory pathways, enhancing Nrf2-associated signaling, and modulating the populations of tumor-associated immune cell. Conversely, testosterone directly promotes tumor growth by triggering inflammatory responses, reducing microbiota diversity, and increasing the proportion of opportunistic pathogens. At the molecular level, there are bidirectional interactions between sex hormones and the gut microbiome. Sex hormones not only regulate the composition and diversity of the gut microbiome but also modulate the host immune response through the gut microbiome. The gut microbiome, in turn, may be involved in the biosynthesis and metabolism of sex hormones by expressing certain enzymes, such as β-glucuronidase and bacterial enzymes. Additionally, the gut microbiota may directly affect gonadal function. However, the available studies present conflicting findings. Some propose that estrogens may increase CRC risk, while androgens seem to confer protection against CRC. Nevertheless, most of these findings are based on clinical samples, and further validation through animal and cellular models is needed.

The prospect of combination therapy, targeting both sex hormones and modulation of the gut microbiome, holds great potential for enhancing outcomes in patients with CRC. Hormonal therapy can be complemented by interventions that modify the gut microbiome composition towards a more protective profile. For example, supplementation of E2 before anti-PD-L1 treatment has been shown to alter the gut microbial composition of MC38 colon tumor-bearing mice, creating a favorable microecological environment and thereby enhancing the anti-tumor efficiency of anti-PD-L1 treatment [92]. On the other hand, manipulating the gut microbiome and its metabolites can also enhance anti-tumor effects. For instance, a combination of anti-PD-L1 with antibiotic colistin treatment significantly reduced testosterone levels in male MC38 mice, leading to increased immunotherapy efficiency [96]. Common methods for modulating the gut microbiome, such as fecal microbiota transplantation and probiotic supplementation, have the potential to modulate sex hormone levels, thereby enhancing the efficiency of immunotherapy [14]. The identification of specific microbial taxa, metabolites, and drugs that augment the antitumor effects of hormonal therapy is an active area of research that paves the way for novel CRC treatment strategies. Future studies should further explore the intricate interactions among sex hormones, the gut microbiome, and host immunity to provide innovative approaches to CRC treatment.

## Conclusion

This review emphasizes the significant influence of sex hormones and the gut microbiome in the development of CRC. Modulating sex hormones or the gut microbiome could have potential clinical implications in preventing and treating CRC. A more thorough comprehension of the interactions among sex hormones, the gut microbiome, and colorectal carcinogenesis could pave the way for novel therapeutic strategies. However, it is important to note that current research in this field is still in its early stages and remains controversial. Further investigation is imperative to gain a deeper understanding of the underlying mechanisms and to optimize therapeutic approaches in this context.

## Data Availability

Not applicable.

## Abbreviations

AOM/DSS: Azoxymethane/dextran sodium sulfate
C/O: Commensal bacteria/opportunistic pathogens
CRC: Colorectal cancer
CSCs: Cancer stem cells
DCA: Deoxycholic acid
DHEA: Dehydroepiandrosterone
DHT: Dihydrotestosterone
E2: Estradiol/17β-estradiol
ERβ: Estrogen receptor- beta
F/B: Firmicutes/Bacteroidetes
FAP: Familial adenomatous polyposis
HSD: Hydroxysteroid dehydrogenase
MDI: Microbial dysbiosis index (MDI)
MDSC: Myeloid-derived suppressor cells
Med/Muc: Medullary/mucinous histology
MMR: Mismatch repair
ORX: Orchiectomy
OVX: Ovariectomy
PGR: Progesterone receptor
SCFAs: Short chain fatty acids
SFB: Segmented filamentous bacteria
SHBG: Sex hormone-binding globulin
TAMs: Tumor-associated macrophages
TIME: Tumor immune microenvironment
TP: Testosterone propionate
UDCA: Ursodeoxycholic acid
UGT: UDP-glucuronosyltransferase
ZEA: Zearalenone
